# Round the Hospitals

**Published:** 1917-06-16

**Authors:** 


### ROUND THE HOSPITALS.

One of the most marvellous changes effected by the war is the whole-hearted way in which our cousins the Americans have thrown themselves into the war for freedom, and justice for the weak. One of the most representative of American hospital authorities and superintendents writes: "The arrival of The Hospital prompts me to send a word of appreciation on behalf of my colleagues and myself. When you were here you found America wavering. Now the die is cast, and the country is slowly, none the less surely, gathering its great strength to apply it where it will accomplish most for the common cause. We hospital men are of course engaged in the task of co-ordinating, expanding, and adapting the hospital and medical resources of the country to meet present and future requirements, and you will be pleased to know that in this connection the columns of The Hospital are proving most helpful." For thirty-two years we have been associated with the leading American hospital men and women, and our visit taught us many things and gave us re-inspiration, especially in connection with the enormous development of the knowledge and influence of women in medicine and hospital work there. We are glad in this connection to reproduce the testimony of Sir Alfred Keogh, Director-General of the Army Medical Service, to a well-known American correspondent: "I wish to emphasise to the [American] organisers the importance of taking the brains of women very seriously. Women have done magnificent work here, and to them none owes a greater debt of gratitude than I do."

---

Miss Lavinia L. Dock, R.N., one of the oldest and ablest workers in the United States, who has interested herself greatly in nurses and nursing in the widest sense, in an article on page 721-2 of the *American Journal of Nursing* for May, demonstrates how one-sided and ill-informed American nursing opinion may be in regard to the nursing position and nursing questions in Great Britain. She states: "In the contest going on in Great Britain over the constitution of the proposed College of Nursing, the Irish nurses have spoken out as a united body, as their wont is in important crises." How opposed to the facts Miss Dock's statement is, the *Weekly Irish Times* of March 10, quoting at length an article by "Ierne," a foremost authority on Irish Nursing, shows: "The entirely unorganised and chaotic condition of nursing in Ireland is nowhere more obvious and deplorable than in the case of the candidate probationer whose plight is particularly unfortunate. Some hospitals charge a fee of fifty guineas for training, others twenty-five, others ten, while one or two are free. These sums, be it observed, form no guide to the status or efficiency of the institution; indeed, in many cases the less expensive are the best. . . . Her hours of duty are long, her duties arduous and responsible, and her conditions of employment in every respect inferior to those of a domestic servant. Perhaps the worst feature of the case is that usually the last person to discover the truth is the probationer herself, who learns only when too late that Dublin hospitals as training-schools do not compare favourably with those of London, Liverpool, or Manchester." A remedy suggested by "Ierne" is: "It would appear as if the establishment in Ireland of the College of Nursing might afford a suitable opportunity for remedying this primitive condition of affairs. The Irish Nurses' Association do not appear to have ever made any effort in the direction of reform, nor indeed to have become properly awake until the College came into being."

---

Miss Dock in her imperfect knowledge proceeds: "As well as can be made out on this side this new scheme (the College of Nursing) so far seems to be mainly another attempt to tie up the whole nursing

ROUND THE HOSPITALS—(*continued*).

profession in England in the grip of the employers and hospital governors. If it evolves differently it will be because of the determined resistance of the clear-sighted ones who are led, need we say, by those faithful watchers and workers, those untiring dynamos of human energy, Mrs. Fenwick and Miss Breay." We regret to find, in view of the facts just stated, that Miss Dock's words are quoted in their entirety in a contemporary as "a chapter of interest by our great nursing historian" without one word of explanation or correction or caution. May we venture to add that we hope steps will be taken in America by American hospital men to enlighten their sisters in the nursing world as to the true facts of the position of British nursing which have been recorded from time to time in the columns of THE HOSPITAL?

THE statement of the claims of the Society for the State Registration of Trained Nurses, in reply to the Memorandum by the College of Nursing Limited, on the Society's petition to the Prime Minister (see THE HOSPITAL of June 2, pages 134 and 136) is a document that will not bear examination by those who know the facts, as the following questions, if they are answered, should clearly demonstrate.

1. On what evidence does the Society dare to declare that the Council of the College is "fighting by every means in its power" against the fundamental principle of the "Direct Representation of Trained Nurses in England and Wales, Scotland and Ireland," on the authority which may be authorised by Parliament "in a Nurses' Registration Act to frame the rules and regulations to which the Registered Nurses will have to conform?"

2. Is not the fundamental principle of the government of the College to be under the amended bye-laws of the constitution, now in process of evolution, more truly democratic and directly representative of the trained nurses in question than that of the Central Committee or the Society itself?

3. Again, how dare the Society declare that the College Council "in the unreasoning fear of all autocrats of liberty for the rank and file has practically forbidden its nurse-members to petition the Prime Minister of this country concerning their own affairs" and their right of government by consent?

4. Is there any justification of any kind for the Society's statement: "The College of Nursing Council is composed of men and women of very reactionary and tyrannical tendencies, whose policy of the suppression of human and professional rights, is a very serious danger to the evolution of the whole nursing profession in this country?"

5. What is the "latest unreasoning act of despotism," on which the Society relies as proof positive of this conviction, and why will it be publicly execrated? when? and by whom?

When we add to these extraordinary and unwarrantable statements the falsehood that each member of the College of Nursing has signed an agreement granting the Council the power to remove her name from the Register, "without power of appeal"—a falsehood because the right to appeal is given in the New Byelaws, as the Society knows full well—and the Society's reply must remain on record as the latest indication of the lengths to which a few ambitious irreconcilables can carry their unreasoning and unworthy attempt to divide and destroy the British Nursing Profession. We hope that this exposure of the true inwardness of one side of the position of nursing affairs in this country to-day may reach and receive the careful and impartial consideration of our American cousins, and so prevent them from sharing the extraordinary misapprehension, through want of knowledge, under which Miss L. L. Dock is labouring at the moment.

THE vacancy caused by the regretted retirement of Miss Amy Hughes from the important office of general superintendent of the Queen Victoria Jubilee Nurses' Institute has been filled by the appointment to that office of Miss A. M. Peterkin. Miss Peterkin's training has extended over a wide field, including her initial training at Chalmers Hospital, Banff, district training at the Metropolitan District Nursing Association, and midwifery at Cheltenham. Miss Peterkin has been associated for twenty-one years with the Queen Victoria Jubilee Institute, in the course of which she has occupied the important positions of inspector for Lancashire and Cheshire and for the Eastern Counties, superintendent for Ireland, and for the past four and a half years superintendent of the Scottish branch of the Institute, a post she now holds. When Miss Amy Hughes undertook the important duty of organising district nursing at the Antipodes, Miss A. M. Peterkin, in reward for her valuable services, was selected and appointed to act as general superintendent of the Institute and its nurses during Miss Hughes' absence. There are few, if any, more arduous posts connected with the British nursing profession than that Miss Peterkin will soon occupy. It is a position which demands full technical knowledge of all branches of nursing, administrative gifts which compel success in great enterprises, and a personality strong enough to win affection and obedience, with the enforcement of authority. In congratulating Miss Peterkin upon her promotion, we wish her the best of health and the completest success both for her own sake and for that of Queen Victoria's Jubilee Institute for Nurses.

MISS C. M. D. CUTHBERTSON has been appointed superintendent of the Drymma Institution for Mental Defectives belonging to the Glamorganshire Joint Poor-Law Establishment Committee. In this connection it is recalled with pardonable pride by the Birmingham Guardians that Miss Cuthbertson is the fourth successive assistant matron from their Monyhull institution to secure a post of matron. The Guardians have promoted Miss E. Hailey, second assistant, to the vacancy of first assistant matron, and Miss G. Langdon, home sister, as second assistant matron.

